# Functional Characterization of *SlSAHH2* in Tomato Fruit Ripening

**DOI:** 10.3389/fpls.2017.01312

**Published:** 2017-07-26

**Authors:** Lu Yang, Guojian Hu, Ning Li, Sidra Habib, Wei Huang, Zhengguo Li

**Affiliations:** School of Life Sciences, Chongqing University Chongqing, China

**Keywords:** tomato, *SlSAHH2*, ethylene, fruit ripening, ACC

## Abstract

S-adenosylhomocysteine hydrolase (SAHH) functions as an enzyme catalyzing the reversible hydrolysis of S-adenosylhomocysteine to homocysteine and adenosine. In the present work we have investigated its role in the ripening process of tomato fruit. Among the three *SlSAHH* genes we demonstrated that *SlSAHH2* was highly accumulated during fruit ripening and strongly responded to ethylene treatment. Over-expression of *SlSAHH2* enhanced SAHH enzymatic activity in tomato fruit development and ripening stages and resulted in a major phenotypic change of reduced ripening time from anthesis to breaker. Consistent with this, the content of lycopene was higher in *SlSAHH2* over-expression lines than in wild-type at the same developmental stage. The expression of two ethylene inducible genes (*E4* and *E8*) and three ethylene biosynthesis genes (*SlACO1, SlACO3* and *SlACS2*) increased to a higher level in *SlSAHH2* over-expression lines at breaker stage, and one transgenic line even produced much more ethylene than wild-type. Although inconsistency in gene expression and ethylene production existed between the two transgenic lines, the transcriptional changes of several important ripening regulators such as *RIN, AP2a, TAGL1, CNR* and *NOR* showed a consistent pattern. It was speculated that the influence of *SlSAHH2* on ethylene production was downstream of the regulation of *SlSAHH2* on these ripening regulator genes. The over-expressing lines displayed higher sensitivity to ethylene in both fruit and non-fruit tissues. Ethylene precursor 1-aminocyclopropane-1-carboxylic acid (ACC) treatment accelerated ripening faster in *SlSAHH2* over-expressing fruit than in wild-type. Additionally, seedlings of transgenic lines displayed shorter hypocotyls and roots in ethylene triple response assay. In conclusion, *SlSAHH2* played an important role in tomato fruit ripening.

## Introduction

Tomato (*Solanum lycopersicum* L.) is one of the most important horticultural crops supplying vitamins and nutrition for human throughout the world. During fruit ripening, color, texture, flavor as well as nutritional status of fruit change and seeds are dispersed (Prasanna et al., 2007; Rugkong et al., 2011). To uncover the mechanism of tomato fruit ripening, lots of ripening-deficient mutants, such as ripening inhibitor (*rin*), never ripe (*Nr*), non-ripening (*nor*), and color non-ripening (*cnr*) have been studied so far (Tigchelaar et al., 1973; Mizrahi et al., 1982; Wilkinson et al., 1995; Vrebalov et al., 2002). Tomato has been classified as climacteric fruit, showing increased ethylene production at or just before the onset of ripening and requiring ethylene to complete the ripening process (Alexander and Grierson, 2002; Paul et al., 2012). *E4* and *E8* are two well known ethylene responsive genes. Suppression the expression of *E4* blocks fruit ripening by inhibiting ethylene biosynthesis (Tigchelaar et al., 1978), and *E8* can influence ethylene biosynthesis in both flowering and fruiting stage (Kneissl and Deikman, 1996). For ethylene biosynthetic pathway, S-adenosylmethionine (SAM) is the substrate; 1-aminocyclopropane-1-carboxylic acid (ACC) synthase (ACS) and ACC oxidase (ACO) are two rate-limiting enzymes (Adams and Yang, 1979; Prasanna et al., 2007). ACS catalyzes the conversion of SAM to ACC while ACO catalyzes the conversion of ACC to ethylene (Murr and Yang, 1975; Boller et al., 1979; Hoffman et al., 1982; Hamilton et al., 1991; Kende, 1993). In tomato, the predominant *SlACS* transcript is *SlACS2* because repression of *SlACS2* blocks ripening and produces unripened fruit (Oeller et al., 1991; Lincoln et al., 1993). *SlACO1* and *SlACO3* are most related to ripening among *SlACO* genes because these two genes can trigger ripening process (Barry et al., 1996; Nakatsuka et al., 1998; Alexander and Grierson, 2002). Tomato fruit color changes by degradation of chlorophyll and accumulation of carotenoids during ripening. Indeed, lycopene is one of the major carotenoids providing red color for tomato fruit (Ronen et al., 1999).

S-adenosylhomocysteine hydrolase (SAHH) is a widespread enzyme in cells. It catalyzes the reversible hydrolysis of S-adenosylhomocysteine (SAH) to adenosine (Ado) and homocysteine (Hcy) (Palmer and Abeles, 1979). The chemical structure of SAH is similar to SAM which is the donor for transmethylation and substrate for ethylene biosynthesis. Hence, SAH is a potential inhibitor of SAM-dependent transmethylation reactions (Chiang et al., 1996; Chiang, 1998; Hermes et al., 2004). SAHH can release the SAH-caused feedback inhibition by catalyzing the hydrolysis of SAH (Weretilnyk et al., 2001). Therefore, SAHH is considered to be essential in regulating the intracellular SAM/SAH ratio. In animals, the inhibition of SAHH enzymatic activity can decrease the replication ability of virus (Matthews et al., 2009). In plants, the biological function of SAHH has also been investigated in many species. *AtSAHH1* and *AtSAHH2* encoding two SAHH enzymes were identified in *Arabidopsis* (Rocha et al., 2005; Pereira et al., 2007; Li C.H. et al., 2008). *AtSAHH1* seemed to be more important because partial loss of *AtSAHH1* showed plant developmental abnormalities. Also, only mutations in *AtSAHH1* were embryonic lethal though sequences between the two *AtSAHH* genes shared higher than 80% identity (Furner et al., 1998; Rocha et al., 2005). Transgenic tobacco expressing antisense RNA of *NtSAHH* was stunted, dwarf, absent of apical dominance and seized of floral abnormalities (Tanaka et al., 1997; Fulneček et al., 2011). Co-silencing of *SlSAHH* genes by VIGS (virus induced gene silencing) in tomato increased immunity to Pst DC3000 and enhanced tolerance to drought stress (Li et al., 2015). SAHH was proposed to be a cytokinin-binding protein and down-regulation of the corresponding gene affected plant morphology by regulating cytokinin pathway and transmethylation cycle (Mitsui et al., 1993; Li C.H. et al., 2008). So far, no information is available on the role of SAHH in fruit ripening. In this paper we ought to uncover the role of SAHH in the ripening process of tomato which is a model plant for studying fruit ripening due to the relatively small genome and ease of genetic manipulation.

In tomato, SAHH is encoded with a gene family containing three members, *SlSAHH1* (Solyc12g098500), *SlSAHH2* (Solyc09g092380), and *SlSAHH3* (Solyc09g092390), with high sequence identity and functional redundancy (Li et al., 2015). A cDNA microarray analysis displayed transcriptional up-regulation of SGN-U314915 (corresponding to *SlSAHH2*) during fruit ripening and complete repression by 1-MCP treatment in tomato (Yan et al., 2013), suggesting a link between SlSAHH proteins and fruit ripening. Our data showed that over-expressing of *SlSAHH2* in tomato accelerated fruit ripening. This was quite different from the reported functions related to plant growth and stress response, thus supporting the hypothesis that SAHH had a significant impact on tomato fruit ripening.

## Materials and Methods

### Plant Materials and Growth Conditions

Tomato (*Solanum lycopersicum* L. cv. Micro-Tom) plants and transgenic lines in this background were grown in a greenhouse under standard conditions (16 h/8 h light/dark cycle, 80% relative humidity, 25˚C/18˚C day/night temperature). Blooming flowers were tagged and ripening stages were divided according to days post anthesis (DPA) and fruit color. For RNA extraction, pigment measurement and SAHH enzyme activity analyses, samples were immediately frozen in liquid nitrogen and then stored at -80˚C until further use. For ACC treatment and ethylene measurements, fresh material was used.

### Expression Pattern of *SlSAHH* Gene Family in Different Tissues

For expression pattern analysis, roots (R), stems (S), leaves (L), floral buds (B), flowers (FL), 16 DPA fruit, mature green fruit (MG), breaker fruit (Br), 2 days post breaker fruit (Br+2) and red ripe fruit (RR) were collected with liquid nitrogen and stored at -80˚C. Due to the high sequence identity and potential functional redundancy of *SlSAHH* genes (Li et al., 2015), primers were designed at 3′ terminal less conserved region of each cDNA. The sequences of primers were listed in **Supplementary Table S1**.

### Expression of *SlSAHH* Genes Responded to Different Hormones

To investigate the expression of *SlSAHH* genes responded to different hormones, tomato fruits at mature green stage (MG, 35 DPA) were picked out. 100 μM ACC, 100 μM IAA, 100 μM GA3, 100 μM ABA or 0.4% ethephon in buffer solution was prepared in turn and hormone injection experiment was performed afterward (Orzaez et al., 2006; Yang et al., 2017). After treatment for 96 h, samples for each treatment were frozen in liquid nitrogen immediately and stored at -80˚C until RNA extraction.

### Construction of Vector pLP100-35S-SAHH2 and Tomato Transformation

ORF sequence of *SlSAHH2* was downloaded from Sol Genomics Network^1^ and amplified with primers SAHH2-F and SAHH2-R (listed in **Supplementary Table S1**). Afterward, plant binary vector pLP100 was chosen to construct over-expression vector pLP100-35S-SAHH2. *Agrobacterium tumefaciens* strain GV3101 was prepared for tomato transformation (Fillatti et al., 1987). The positive transgenic lines were screened by kanamycin (100 mg⋅L^-1^) selection and GUS staining. After qPCR confirmation, two successful over-expression lines (OE-5# and OE-6#) in T2 and T3 generation were selected for further analysis.

### Recombinant Expression and Purification of SAHH2

A mature protein coding region of SAHH2 was amplified with primers listed in **Supplementary Table S1** and then subcloned into pET-28a (+) vector (Novagen, Darmstadt, Germany) between the *Sac I*- and *BamH I*- sites with the poly-histidine at N-terminal. After that, a single colony of *Escherichia coli* BL21 (DE3) harboring the pET28a-SAHH2 vector was cultured in 5 mL LB medium with 100 μg⋅mL^-1^ kanamycin and grown overnight at 37˚C. This culture was next extended to 100 mL shaken at 37˚C with 250 rpm until OD600 reached 0.6–0.8. Collect 5 mL of the bacteria liquid referred as 0 h and then add IPTG (CWBIO, China) into the left culture with a final concentration of 1 mM. Subsequently, the culture was shaken at 180 rpm at 30˚C for additional 2 h, 4 h, and 6 h. Bacteria liquid was collected by centrifugation at 4000 *g* for 10 min at 4˚C, washed twice and resuspended with 20 mM PB buffer (phosphate buffer, pH7.4). Then the solution of bacteria cells supplemented with 1 mg⋅mL^-1^ lysozyme and 1 mM PMSF was sonicated on ice until the cell lysate clarified. At last, the supernatant after centrifugation and pellet resuspended with PB of different time points were all used to run SDS–PAGE. For purification of the recombinant protein SAHH2, a Ni-Agarose Kit for His tag soluble protein (CWBIO, China) was used with some modifications.

### SAHH Enzymatic Activity Measurement

Measurement of SAHH activity was carried out following published methods (Wolfson et al., 1986), with slight modifications. *In vitro* experiment, the purified 5, 10, 15, 20 and 25 μg recombinant protein removing salts and other small molecules through Sephadex G25 columns were used to assay the hydrolysis activities. Reactions were conducted at 25˚C for 15 min in 1 mL reaction mixture contained: 50 mM HEPES-KOH (pH 7.8), 1 mM EDTA (pH 8.0), 0.1 mM DTNB (Sigma), 0.1 mM SAH (Sigma), and required dose of recombinant protein SlSAHH2. In the reaction assay, Hcy was used as a reducing reagent for 5, 5′dithiobis-(2-nitrobenzoic acid) (DTNB) to DTNB-thiolate, which resulted in an increase in the absorbance of the reaction mixture at 412 nm. *In vivo* experiment, 100 mg tissues from WT and transgenic lines at different stages were ground in ice-cold HEPES buffer (pH7.8, 50 mM HEPES, 5 mM DTT, 1 mM Na_2_EDTA, 5 mM ascorbic acid, 10 mM boric acid, 20 mM Na-metabisulfate and 4% Polyvinylpyrrolidone) and extracts were collected by centrifugation at 4˚C for 5 min. The supernatant (0.5 mg) was used for enzyme activity assays. The experiments were performed in three repetitions with six replicates each.

### Determination of Fruit Ripening Time

Tomato flowers were tagged and fruit ripening time was observed. On-vine ripening period was expressed as the number of days needed from anthesis to breaker (DPA). The experiment was carried out with nine individual plants for each line and repeated for three generations (T1–T3).

### Measurement of Ethylene Production

In order to determine ethylene production level, pre-weighed fruits at breaker stage were harvested and placed in open 50 mL air-tight containers for 3 h to avoid the effect of ethylene emission caused by picking. Jars were then sealed with paraffin wax and incubated at room temperate for 16 h. Afterward, 1 mL of headspace gas was injected into a gas chromatograph (Varian CP-3800 GC gas chromatograph, United States) fitted with an activated alumina column and a flame ionization detector. Reagent-grade ethylene standards were used for evaluating ethylene content and ethylene production in fruit was calculated with normalization of fruit weight (Chung et al., 2010). Three biological replicates were adopted and each replicate contained at least 10 fruits.

### Lycopene Extraction

Five gram fruit pericarp at 43- and 46- DPA were prepared in a beaker. Little amount of methanol was added into the beaker and then stirred with a glass rod adequately. After that, filtered the solution with filter paper and repeated the above steps until the extract became colorless. Next, discard the extraction and extract the left residue with little amount of petroleum ether several times until the extraction became colorless again. Collect the extraction which was the tomato red pigment extract for following experiment. For standard curve drawing, different concentration of the sultan I solution was used. For lycopene content measurement, 1–2 ml extractions together with absolute alcohol supplied was used. The UV spectra were monitored at 485 nm. Two independent experiments were performed as biological replicate for each sample with three technological replicates.

### ACC Treatment of Tomato Fruit

For ACC treatment experiment, tomato fruit were harvested at breaker stage and injected with a buffer solution (pH 5.6) contained MES (10 mM), sorbitol (3% w/v) and ACC (100 μM) as described above. Briefly, fruits were injected with a 1 ml syringe containing a 0.5 mm needle, inserted 3–4 mm into the fruit tissue from the stylar apex. The solution was gently injected into the fruit until the buffer ran off the hydathodes and stylar apex at the tip of the sepals. Only completely infiltrated fruits were used for next experiments. Then fruits were incubated in a culture room at 26˚C, under 16 h light/8 h dark cycles with light intensity of 100 μmol m^-2^ s^-1^. After 96 h, the difference of changes in color was observed and fruits pericarps were frozen at -80˚C until further analysis.

### Ethylene Triple Response Assay

For ethylene triple response assay, seeds of WT and transgenic lines were sterilized and sown on MS medium with 1 μM ACC or not. Then the seeds were all cultured at 25˚C in the dark. Root and Hypocotyl elongation were observed and measured 7 days post sowing. For each line, at least 30 seedlings were measured. To clarify the molecular mechanism, the expression of *E4, E8, ACO1, ACO3* and *ACS2* was detected by real-time PCR.

### RNA Extraction and Real-Time PCR Analysis

For each line, three independent biological replicates were used and six fruits collected from different plants were referred to as one sample. Total RNAs of samples were extracted using Trizol (Invitrogen) according to the manufacturer’s instructions. Reverse transcription of the first-strand cDNA was performed with RevertAid^TM^ First Strand cDNA Synthesis Kit (Fermentas, United Kingdom). Gene-specific primers were designed with the software of Primer Express 5.0 and *SlActin* (Solyc03g078400) was used as internal control. QPCR was performed using the SyBR Green PCR Master Mix (CWBIO, China) in a 25 μL total sample volume (1.0 μL of cDNA, 1.0 μL of primers, 12.5 μL of 2×SYBR Mix Taq and 10.5 μL of distilled water). Reaction was performed with an initial incubation at 95˚C for 20 s, followed by 40 cycles of 95˚C for 3 s and 60˚C for 30 s with Bio-Rad CFX connect (Bio-Rad, United States). The cycle threshold (Ct) 2^-Δ(ΔCt)^ method was adopted for relative quantification the specific mRNA levels (Livak and Schmittgen, 2001). Primers used for real-time PCR were all shown in **Supplementary Table S1**.

### Statistical Analysis

All experiments were repeated three times independently and all results were reproducible. Statistical results were presented as means ± standard error. To compare group differences, two-tailed Student’s *t*-tests were used. *P*-values less than 0.05 were recognized as significant.

## Results

### Gene Expression Pattern Analysis of *SlSAHH* Gene Family

**FIGURE 1:**
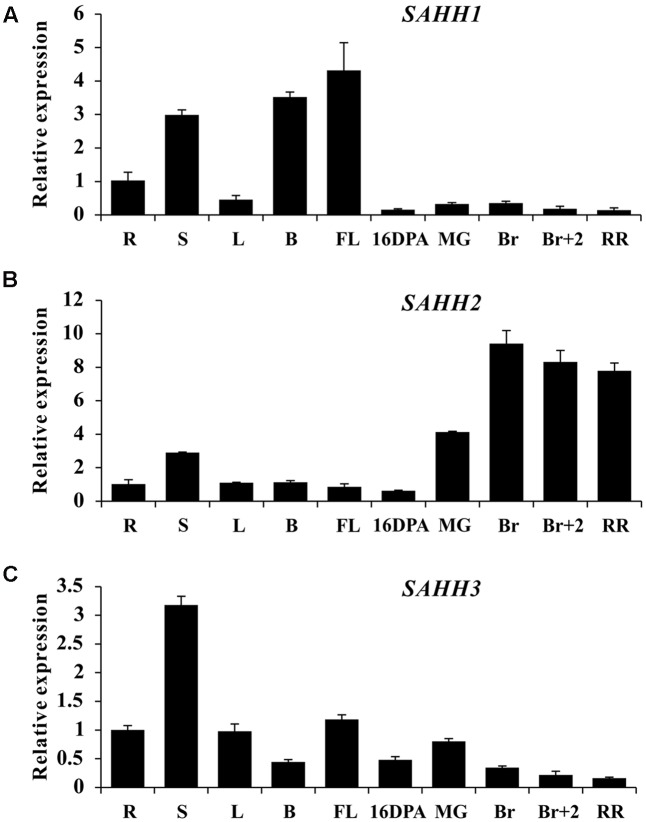
Expression patterns of *SlSAHH* genes in wild-type tomato (WT). **(A)** Tissue profile analysis of *SlSAHH1* in different tissues in WT. **(B)** Tissue profile analysis of *SlSAHH2* in different tissues in WT. **(C)** Tissue profile analysis of *SlSAHH3* in different tissues in WT. R, Roots; S, stems; L, leaves; B, floral buds, FL, anthesis flowers; 16 DPA, 16 days post anthesis; MG, mature green fruit; Br, color breaker fruit; Br+2, 2 days post breaker fruit; RR, red ripe fruit. The quantitative PCR data represent mean values for three independent biological replicates (*n* = 3).

To explore the expression profile of *SlSAHH* gene family, real-time PCR was performed in roots (R), stems (S), leaves (L), buds (B), flowers (FL), and fruit at different ripening stages of wild-type tomato. Although functional redundancy existed among the three genes (Li et al., 2015), their expression patterns were quite different. *SlSAHH1* was highly expressed in stem, buds and flowers, with a rapid decline in all fruit tissues (**Figure 1A**). Interestingly, *SlSAHH2* showed quite different expression pattern. The mRNA level was lower in roots, stems, leaves, buds, flowers and 16DPA fruit, while highly accumulated during fruit ripening especially at breaker stage (**Figure 1B**). Additionally, the expression profile of *SlSAHH3* was distinct from the first two members. High transcriptional level can be seen in the tissues of roots, leaves and flowers and the maximum expression level was displayed in stem, but its transcriptional level was lower in ripe fruit (**Figure 1C**). These results indicated that the expression patterns of the three *SlSAHH* genes were all different and *SlSAHH2* may function in the process of fruit ripening.

### *SlSAHH* Family Genes Are Regulated by Various Phytohormones

**FIGURE 2:**
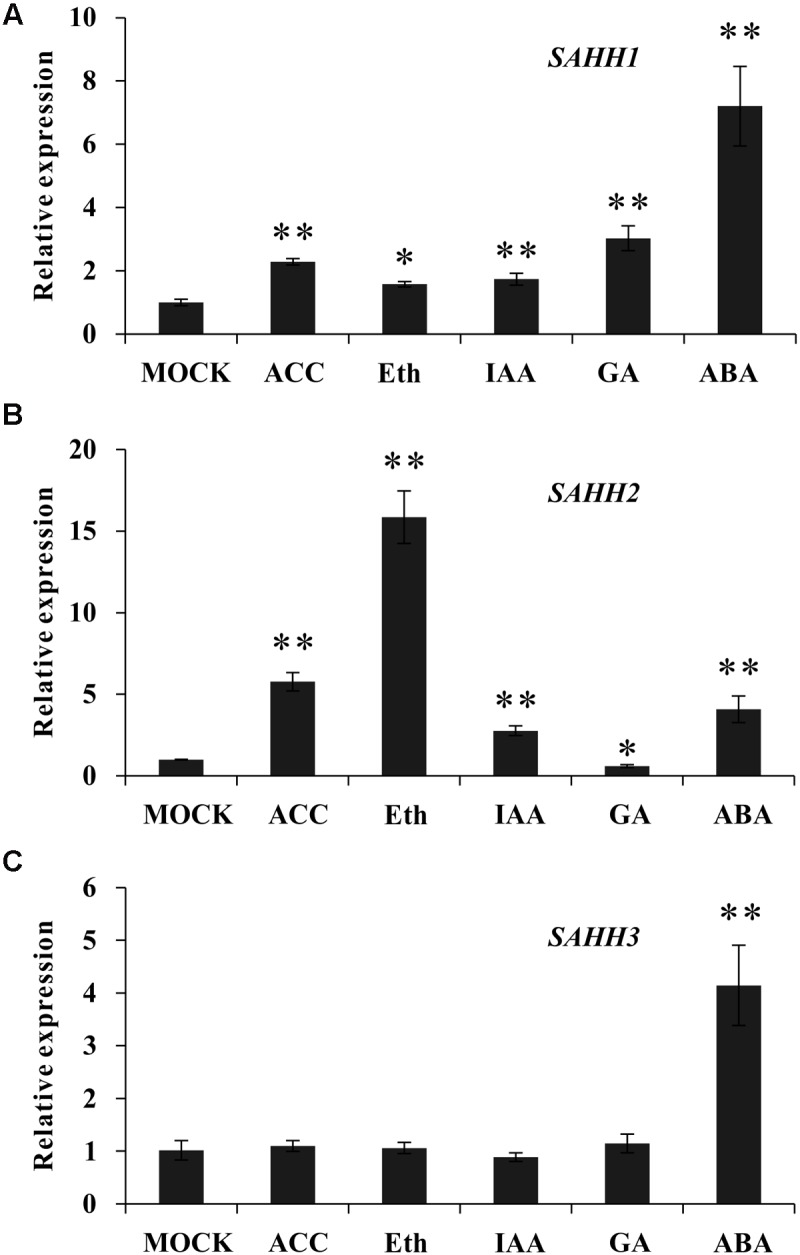
The response of *SlSAHH* gene family members to different types of ripening related hormones after treatment for 96 h. **(A)** The expression of *SlSAHH1* responded to phytohormones. **(B)** The expression of *SlSAHH2* responded to phytohormones. **(C)** The expression of *SlSAHH3* responded to phytohormones. Mock, solution (pH 5.6) contained 10 mM MES and sorbitol (3% w/v); ACC, 1-aminocyclopropane-1-carboxylic acid; Eth, ethephon; IAA, indole-3-acetic acid; ABA, abscisic acid. The quantitative PCR data represent mean values for three independent biological replicates (*n* = 3). As determined by *t*-test, ^∗^ and ^∗∗^ indicate significant differences between mock and hormone treated group with *P* < 0.05 and *P* < 0.01, respectively.

To determine whether the expression of *SlSAHH* gene family members could be regulated by phytohormone, qPCR was conducted with wild-type MG fruit treated by exogenous ethephon, ACC, GA3, IAA and ABA. As shown in **Figure 2A**, the expression of *SlSAHH1* was induced by all these hormones treatment. The mRNA level of *SlSAHH3* changed little upon ethephon, ACC, GA3 and IAA treatment. Similarly, both *SlSAHH1* and *SlSAHH3* were induced significantly after ABA treatment (**Figure 2C**). Interestingly, the mRNA level of *SlSAHH2* increased significantly (16-folds) with the stimulation of exogenous ethylene. Also, the expression of *SlSAHH2* can be up-regulated by IAA and ABA and down-regulated by GA3 (**Figure 2B**). These findings indicated that *SlSAHH* family genes could be regulated by phytohormones and the expression of *SlSAHH2* was strongly induced by ethylene.

### Over Expression of *SlSAHH2* Enhances SAHH Enzymatic Activity

**FIGURE 3:**
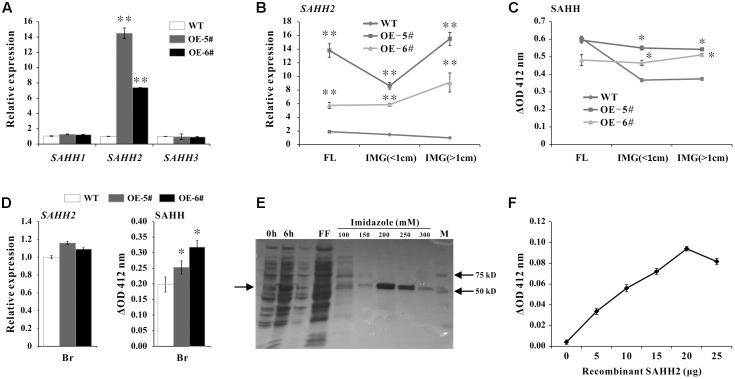
Over expression of *SAHH2* enhanced SAHH enzymatic activity. **(A)** The mRNA levels of *SlSAHH1, SlSAHH2* and *SlSAHH3* in leaves of WT and transgenic lines. WT, wild-type; OE-5# and OE-6#, two independent *SlSAHH2* over-expression (OE) lines. **(B)** The mRNA level of *SlSAHH2* in FL and IMG fruit of WT and transgenic lines. FL, flowers; IMG (<1 cm), immature fruit (diameter less than 1 cm); IMG (>1 cm), immature fruit (diameter more than 1 cm). **(C)** SAHH enzymatic activity analysis in FL and IMG fruit of WT and transgenic lines. **(D)** The mRNA level of *SlSAHH2* and corresponding SAHH enzymatic activity in WT and transgenic breaker fruit. **(E)** Recombinant expression and purification of *SlSAHH2*. 0 h, sample collected without IPTG induction; 6 h, sample collected 6 h after IPTG induction; FF, flow-through fraction; 100, 150, 200, 250, 300 refer to different concentration of imidazole (mM) used for elution. **(F)** Enzyme activity detection with the gradually increased dose of recombinant protein SAHH2. The quantitative PCR data represent mean values for three independent biological replicates (*n* = 3). ^∗∗^Refers to significant differences between transgenic lines and WT with *P* < 0.01, as determined by *t*-test. The SAHH enzymatic activity assays were performed in three repetitions with six replicates each. ^∗^Refers to significant differences between transgenic lines and WT with *P* < 0.05, as determined by *t*-test.

To further investigate the function of *SlSAHH2*, transgenic tomato lines were created by over expressing *SlSAHH2* using its full-length cDNA with pLP100 vector under CaMV 35S promoter. WT and five different transgenic lines were grown under the same condition. For qPCR analysis in leaves, *SlSAHH2* mRNA level was up-regulated significantly in two independent lines (OE-5# and OE-6#) without affecting the expression of other two homologous genes (**Figure 3A**). To establish the correlation between *SlSAHH2* and ripening time, the expression of *SlSAHH2* and its corresponding SAHH enzyme activity were detected in flower and early development fruit. According to the higher mRNA level of *SlSAHH2* in transgenic lines (**Figure 3B**), SAHH enzyme activity enhanced about 20–30% in transgenic IMG fruit than in WT (**Figure 3C**). However, SAHH enzyme activity showed no significant change in transgenic flower than in WT (**Figure 3C**). Also, SAHH enzyme activity remained higher in transgenic breaker fruit although the *SlSAHH2* mRNA level showed no significant change compared to WT (**Figure 3D**). So the inconsistency between mRNA level and enzyme activity existed. To give strong evidence that *SlSAHH2* can functioned as an enzyme, recombinant protein SlSAHH2 (theoretical molecular weight: 56.48 kDa) was obtained in *E. coli*. It was induced strongly by IPTG at 6 h in supernatant and the optimized concentration of imidazole for purification was 200 mM (**Figure 3E** and **Supplementary Figure S1**). The enzyme activity enhanced nearly linearly in a dose-dependent way of SAHH2, which indicated that SlSAHH2 can hydrolyze substrate *in vitro* (**Figure 3F**).

### *SlSAHH2* Impacts Fruit Ripening

**FIGURE 4:**
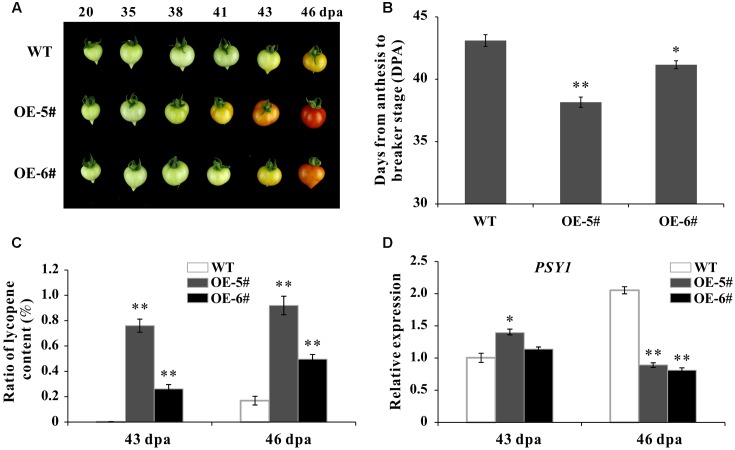
Phenotypic characterization of wild-type and transgenic plants. **(A)** Phenotype of *SlSAHH2* over expression fruit (OE-5# and OE-6#). The OE fruit color changed earlier than WT in the process of ripening. **(B)** Days from anthesis to breaker stage in WT and transgenic fruit. Calculation was carried out with nine individual plants for each line and repeated for three generations (T1–T3). ^∗^ and ^∗∗^ indicate *P* < 0.05 and *P* < 0.01, respectively. **(C)** Analysis of lycopene accumulation at 43- and 46-DPA of transgenic fruit and WT. Standard error is indicated for a minimum of three fruits per sample. ^∗∗^Refers to significant differences between transgenic and WT plants with *P* < 0.01, as determined by *t*-test. **(D)** Expression of *PSY1* in 43- and 46-DPA fruit of transgenic lines and WT. The quantitative PCR data represent mean values for three independent biological replicates (*n* = 3). ^∗^ and ^∗∗^ indicate *P* < 0.05 and *P* < 0.01, respectively.

During fruit development, the time from blossom to breaker stage was calculated (DPA) by tagging the flowers. In present study, the whole ripening time was shortened in OE fruit than in WT. For instance, at 41 DPA, the WT fruit was still be mature green while the OE-5# fruit was in Br+3 and the OE-6# fruit was in breaker stage (**Figure 4A**). The ripening process was significantly accelerated in OE-5# (about 5 days) and OE-6# (about 2 days) than in WT (**Figure 4B**). As the main content of carotenoids, lycopene content was extracted and determined. The accumulation of lycopene in OE fruit was much higher at both 43- and 46-DPA (**Figure 4C**). Phytoene synthase 1 plays a role in rate-limiting step of carotenoid synthesis during tomato fruit ripening. Consistent with lycopene content, the expression of Phytoene synthetase1 coding gene *SlPSY1* was up-regulated in OE fruit at 43 DPA (**Figure 4D**). The decreased expression of *SlPSY1* existed in OE fruit at 46 DPA may be because of negative feedback. So *SlSAHH2* was accounting for enhanced lycopene in ripening fruit.

### Changes in the Expression of Ripening and Ethylene-Related Genes in OE Fruit

**FIGURE 5:**
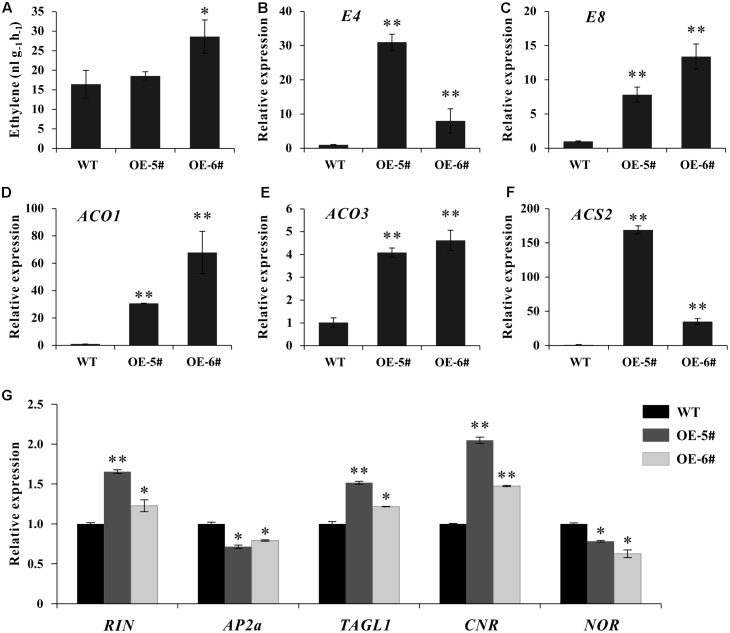
Analysis of ethylene production and expression of ethylene related genes in OE-5#, OE-6# and WT fruit at breaker stage (Br). **(A)** Production of ethylene in WT and OE fruit. Fresh breaker fruit were sealed in air-tight vials, and 1 mL of gas was sampled from the headspace after 16 h. Values represent means from 10 individual fruits. Error bars represent SE. **(B)** Expression of *E4* in OE lines and WT in breaker fruit. **(C)** Expression of *E8* in OE lines and WT in breaker fruit. **(D)** Expression of *ACO1* in OE lines and WT in breaker fruit. **(E)** Expression of *ACO3* in OE lines and WT in breaker fruit. **(F)** Expression of *ACS2* in OE lines and WT in breaker fruit. **(G)** Expression of *RIN, AP2a, TAGL1, CNR* and *NOR* in OE lines and WT in breaker fruit. For qPCR analysis, the data represent mean values for three independent biological replicates. ^∗^ and ^∗∗^ indicate significant differences between transgenic lines and WT with *P* < 0.05 and *P* < 0.01, respectively, as determined by *t*-test.

Considering tomato fruit ripening was predominantly controlled by ethylene, the expression of several ripening regulators and ethylene related genes was detected. At breaker stage, although ethylene production just increased significantly in OE-6# transgenic line (**Figure 5A**), the expression of two ethylene inducible genes (*E4* and *E8*) and three ethylene biosynthesis genes (*SlACO1, SlACO3* and *SlACS2*) increased significantly in both two transgenic lines (**Figures 5D–F**). Additionally, at early fruit developmental stages of transgenic lines, the expression of these genes was initially suppressed in flower and then began to increase in IMG fruit (**Supplementary Figure S2**), displaying the gradually enhanced expression pattern with fruit development and ripening. The transcriptional levels of several important ripening regulators were also influenced as expected. The expression of *RIN, TAGL1* and *CNR* were up-regulated. As a negative regulator, *AP2a* was inhibited in *SlSAHH2-*OE fruit. At the same time, *NOR* was also down-regulated because it was a positive regulator of *AP2a* (**Figure 5G**). The results suggested that over-expression of *SlSAHH2* in tomato influenced the expression of ripening regulators and ethylene related genes significantly, which even enhanced ethylene production.

### The OE Fruit Is Sensitive to Ethylene

**FIGURE 6:**
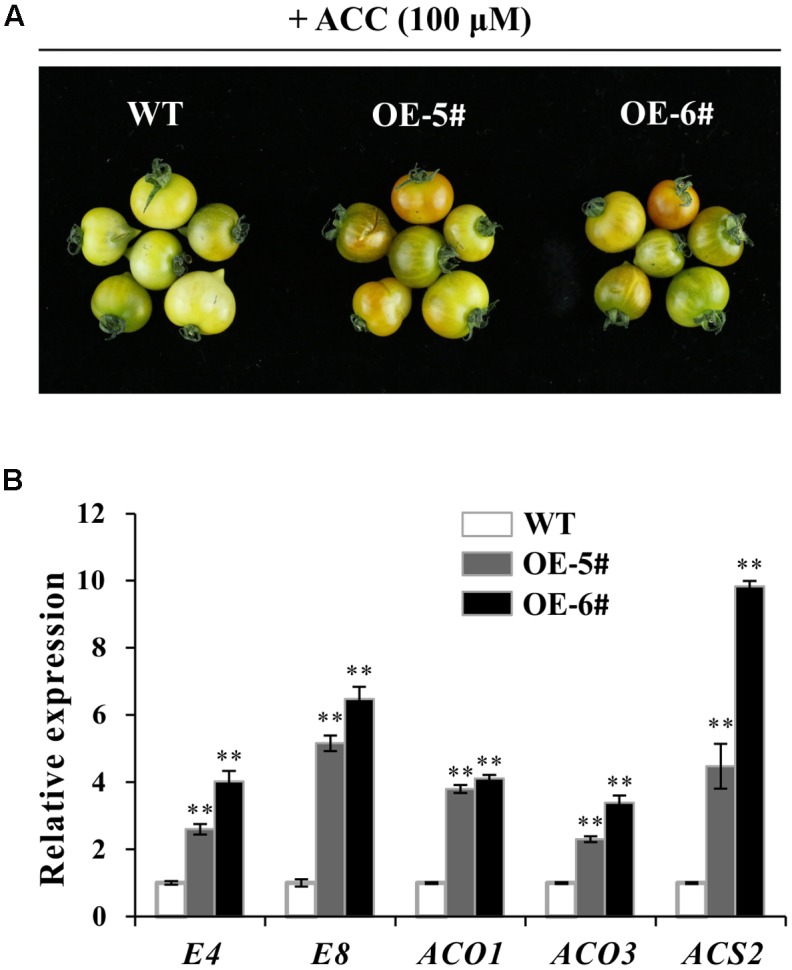
ACC treatment showed a more obvious effect in accelerating ripening in transgenic fruit. **(A)** More obvious color change in OE fruits after ACC treatment for 96 h. **(B)** Relative expression of ethylene and ripening relate gene (*E4, E8, ACO1, ACO3* and *ACS2*) in WT and OE fruit after ACC treatment for 96 h. The quantitative PCR data represent mean values for three independent biological replicates (*n* = 3). ^∗∗^Refers to significant differences between transgenic lines and WT with *P* < 0.01 determined by *t*-test.

Fruit ripening was a process of being sensitive to ethylene. To investigate ethylene sensitivity in WT and transgenic fruit, ethylene precursor 1-aminocyclopropane-1-carboxylate (ACC) was used for treatment. After treatment for 96 h, color of OE fruit pericarp turned much more quickly (**Figure 6A**). When fruit became a little orange in OE-5# and OE-6#, the color in WT fruit was still pale yellow. These results indicated that pigmentation of OE fruit was partly dependent on ethylene. It was concluded that OE fruit was much more sensitive to ethylene which can accelerate the process of ripening. QPCR results indicated that *E4, E8, SlACO1, SlACO3* and *SlACS2* were all up-regulated in OE-5# and OE-6# fruit after ACC treatment, which was in accordance with the above observation (**Figure 6B**).

### The OE Seedlings Are Sensitive to Ethylene

**FIGURE 7:**
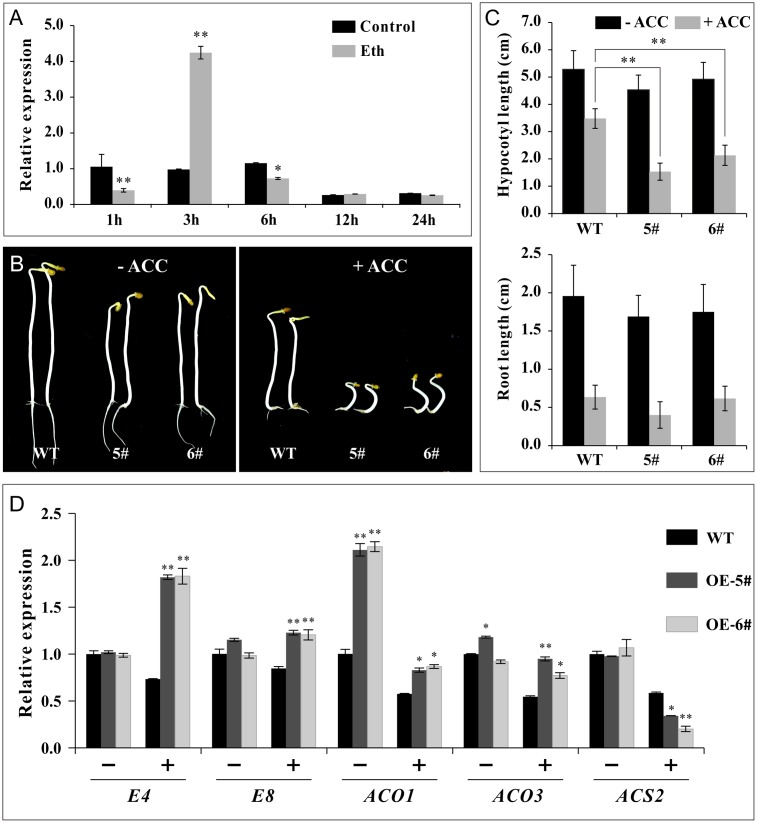
*SlSAHH2-*OE seedlings were sensitive to ethylene. **(A)** The response of *SlSAHH2* to ethylene at different time points in 7 days seedlings of wild-type. Control, half-strength Murashige and Skoog (MS/2) medium; Eth, MS/2 medium supplemented with 20 μM ethephon. 1, 3, 6, 12, and 24 h refer to different time points with ethephon treatment. ^∗^ and ^∗∗^ indicate *P* < 0.05 and *P* < 0.01, respectively. **(B)** Ethylene triple response assay. Seedlings of wild-type (WT) and transgenic lines (OE-5# and OE-6#) treated with 0 (left) and 1.0 μM (right) ACC. **(C)** Statistics of elongation of hypocotyl and root growth after treatment with 0 and 1.0 μM ACC. Error bars represent ± SE. ^∗∗^Refers to significant differences between transgenic lines and WT with *P* < 0.01 determined by *t*-test. **(D)** Expression of ethylene related genes (*E4, E8, ACO1, ACO3* and *ACS2*) in seedlings of WT and OE lines treated with (+) or without (-) 1 μM ACC. The quantitative PCR data represent mean values for three independent biological replicates (*n* = 3). ^∗^ and ^∗∗^ indicate *P* < 0.05 and *P* < 0.01, respectively.

Furthermore, experiments were conducted in non-fruit tissue to confirm ethylene sensitivity. The transcriptional levels of *SlSAHH2* increased substantially at 3 h, while evidently decreased at 1 and 6 h post ethephon treatment in WT seedlings, revealing that the regulation of ethylene on *SlSAHH2* in non-fruit tissue was effective (**Figure 7A**). In ethylene triple response assays, WT, OE-5# and OE-6# seeds were germinated on Murashige and Skoog medium with or without the supplement of ACC. The elongation of roots and hypocotyls was measured 7 days post sowing. It was demonstrated that the average length of transgenic hypocotyls and roots was shorter than WT no matter in the absence (0 μM) or presence (1.0 μM) of ACC (**Figures 7B,C**). We detected the mRNA level of *E4, E8, SlACO1, SlACO3* and *SlACS2* in seedlings with or without ACC treatment. *SlACO1* showed prominent up-regulation in OE-5# and OE-6# before treatment. Except for *SlACS2*, other genes were all up-regulated significantly after ACC treatment (**Figure 7D**). The qPCR results were basically in line with the morphologic changes of seedlings, revealing that the transgenic lines were also sensitive to ethylene in non-fruit tissues.

## Discussion

As a key enzyme in maintaining methylation cycle in cells, SAHH has been investigated in various organisms. In higher plants, it has been proved to play a critical role in plant growth and development (Tanaka et al., 1997; Rocha et al., 2005; Ouyang et al., 2012). Also, it functions as targets of gene silencing suppressors in defending response to many pathogens (Yang et al., 2011; Cañizares et al., 2013; Li et al., 2015). SAHH are encoded by two genes in *Arabidopsis* and three in tomato (Rocha et al., 2005; Li et al., 2015). Silencing of single *SlSAHH* gene does not contribute to any defect in tomato plant growth because of functional redundancy (Li et al., 2015). Here, we found expression patterns of the three *SlSAHH* genes in wild-type tomato were quite different and *SlSAHH2* showed extremely high expression at breaker stage (**Figure 1**). Also, *SlSAHH2* was up-regulated significantly by ethylene treatment in fruit (**Figure 2B**). Similarly, DNA microarray analyses indicated that the transcription of SGN-U314915 (corresponding to *SlSAHH2*) was higher during fruit ripening and lower with 1-MCP treatment in tomato (Yan et al., 2013). This led us to hypothesize that *SlSAHH2* may function during tomato fruit ripening in presence of ethylene being produced.

Although there were other two *SlSAHH* genes in tomato, *SlSAHH2* encoding protein possessed SAHH enzyme activity both *in vivo* and *in vitro* (**Figure 3**). Over-expression of *SlSAHH2* in tomato accelerated fruit ripening about 2–5 days compared to WT, and the whole ripening time was shortened in OE fruit (**Figures 4A,B**). Lycopene represents more than 70% content of carotenoids and contributes to the red color in fully ripe tomato (Fraser et al., 1994; Burns et al., 2003; Alba et al., 2005; Ma et al., 2014). In our study, the content of lycopene was higher in OE fruit than in WT at 43- and 46- DPA, which was consistent with expectation (**Figure 4C**). To reveal the mechanism of color change by molecular evidence, the expression of *SlPSY1* was also detected because PSY1 was the main rate-limiting enzyme of carotenoid biosynthesis (Fraser et al., 1994, 2002; Li F. et al., 2008). Although a decrease in the mRNA level of *SlPSY1* at 46 DPA existed may be due to negative feedback, *SlPSY1* was up-regulated significantly at 43 DPA (**Figure 4D**). These results pointed to the discrepancy between mRNA level and product level.

The accumulation of lycopene in red ripe tomato caused by SlPSY1 is partly mediated by ethylene (Maunders et al., 1987; Fraser et al., 1994; Ronen et al., 1999; Alba et al., 2005). In our study, the expression of *E4, E8, SlACO1, SlACO3* and *SlACS2* was detected in a time-course manner. *E4* and *E8* are two ethylene inducible genes, and ACS and ACO are rate-limiting enzymes for ethylene synthesis (Lincoln et al., 1987; Lincoln and Fischer, 1988; Peñarrubia et al., 1992; Barry et al., 2000). The expression of these genes in transgenic lines was suppressed in flower and began to increase at early fruit developmental stage (**Supplementary Figure S2**). When fruit reached breaker stage, the expression of these five genes increased significantly in transgenic lines (**Figures 5B–F**). Tomato fruit undergoes a developmental transition from ethylene system I to ethylene system II during ripening (Bleecker and Kende, 2000). System I is a process of ethylene auto-inhibitory and System II is a process of ethylene auto-catalytic. Several ethylene related gene such as *ACS2* and *ASC4* can be up-regulated from the transition from system I to system II (Burg and Burg, 1965; Mcmurchie et al., 1972; Klee and Giovannoni, 2011). During fruit ripening, several important transcription factors are considered to be hallmarks and regulators in ethylene dependent or independent manner (e.g., RIN, AP2a, TAGL1, CNR and NOR). As the member of MADS box gene family, RIN binds to the promoter of *ACS2* and plays a role in the upstream regulatory cascade of ethylene (Vrebalov et al., 2002). In our result, the expression of *RIN* was up-regulated in OE breaker fruit (**Figure 5G**). TAGL impacts tomato fruit ripening by inducing autocatalytic ethylene production in system II. Repression of *TAGL1* produces yellow-orange color fruit with lower ethylene content and decreased *ACS2* expression (Bemer et al., 2012; Gimenez et al., 2016). In our study, the expression of *TAGL1* was induced in OE fruit (**Figure 5G**). This was consistent with the enhanced ethylene production phenotype, hinting that *SlSAHH2* may accelerate fruit ripening by increasing the expression of *TAGL1*. In addition, *TAGL1* can be positively regulated by *CNR* (Bemer et al., 2012). In line with *TAGL1*, the transcription level of *CNR* was also higher in OE fruit (**Figure 5G**). In tomato, APETALA2a (AP2a) is a negative regulator of fruit ripening with a negative feedback loop (Chung et al., 2010). It represses ethylene production by inhibiting the expression of ethylene biosynthesis genes. *AP2a*-RNAi transgenic tomato produces more ethylene than WT at the same ripening stages (Karlova et al., 2011). The decreased mRNA level of *AP2a* in *SlSAHH2*-OE fruit also perfectly explained the phenotype of more ethylene production. As a positive ripening regulator of AP2a (Chung et al., 2010), the expression of *NOR* was down-regulated at the same time (**Figure 5G**).

In plants, SAM is the important substrate for ethylene biosynthesis and donor for transmethylation reactions. It can be produced again through efficient recycling of MTA and Hcy (Van de Poel et al., 2013). SAHH removes negative function of SAH to guarantee adequate SAM supplement and high amount of SAM is available during fruit ripening (Moffatt and Weretilnyk, 2001; Van de Poel et al., 2013). It was speculated that *SlSAHH2* may accelerate tomato fruit ripening indirectly by influencing SAM content. Although the phenotypes in the two transgenic lines were similar, inconsistency also existed between OE-5# and OE-6#. For example, the mRNA level of *SlSAHH2* was higher in OE-5# than in OE-6#, but the expression of most ethylene biosynthetic and responsive genes was higher in OE-6# (**Figures 5C–E**). Also, ethylene production increased significantly in OE-6# rather than in OE-5# (**Figure 5A**). Nevertheless, the expression patterns of ripening regulator genes were consistent with the expression of *SlSAHH2* in these two lines (**Figure 5G**). It was speculated that the influence of *SlSAHH2* on ethylene production was probably downstream of the regulation of *SlSAHH2* on the major ripening genes, and the inconsistency between the expression of *SlSAHH2* and ethylene biosynthesis in OE-5# and OE-6# was probably due to feedback regulation.

Ethylene accelerates chlorophyll degradation and lycopene accumulation in fruit (Egea et al., 2011). As the precursor of ethylene biosynthesis, ACC can induce significant color changes within 96 h and accelerate the color transition from green to orange/red (Su et al., 2015). Fruit ripening is a process of being sensitive to ethylene because ethylene-insensitive plants such as the Never-ripe (Nr) mutant exhibits non-ripening phenotype due to failing to respond to the high ethylene levels (Wilkinson et al., 1995). With the treatment of ACC, Our results showed the transgenic fruit can even ripen faster than WT (**Figure 6A**). Moreover, ethylene related genes were all up-regulated (**Figure 6B**). These results revealed that over expression of *SlSAHH2* enhanced ethylene sensitivity of tomato fruit during ripening. To confirm the conclusion in non-fruit tissues, ethylene response of seedlings was also detected. In the triple response assays, the hypocotyl and root elongation of transgenic seedlings were shorter than WT with or without ACC treatment (**Figure 7**). This result indicated that the transgenic seedlings contained more endogenous ethylene and showed sensitivity to exogenous ethylene. QPCR results were consistent with the phenotype except the expression of *SlACS2*. One probable speculation was that *SlACS2* mainly functioned on transiting system I to system II in fruit tissue (Nakatsuka et al., 1998).

DNA hypomethylation is associated with SAHH silencing in plants on account of SAHH can release the SAH-caused feedback inhibition and promote further continual transmethylation reactions (Mull et al., 2006; Jordan et al., 2007; Ouyang et al., 2012). Previously investigation in WT and tomato ripening mutants suggested that tomato fruit ripening was a process of DNA-hypomethylation (Manning et al., 2006; Zhong et al., 2013). Our qPCR results revealed that DNA methyltransferases genes *SlDRM5, SlDRM7, SlDRM8* and *SlMET1* were all up-regulated at breaker stage in transgenic fruit (**Supplementary Figure S3**). However, the methylation state of ripening-related genes and the regulation between ethylene and DNA methylation resulting from the *SlSAHH2*-OE fruit was still unknown. So explanation of the mechanism is in dire need of further investigations.

In summary, over-expression of *SlSAHH2* impacts the expression of ripening related genes, changes ethylene sensitivity, and accelerates tomato fruit ripening. Although detailed regulatory cascade remains to be discovered, this report provides new insights of the role of *SlSAHH2* in fleshy fruit ripening.

## Author Contributions

LY performed research and wrote the manuscript. ZL directed research. NL and WH supplied helps in some experiments. GH and SH supplied helps in revising the manuscript.

## Conflict of Interest Statement

The authors declare that the research was conducted in the absence of any commercial or financial relationships that could be construed as a potential conflict of interest.

## Footnotes

1 https://solgenomics.net/

## Supplementary Material

FIGURE S1 Recombinant expression of *SlSAHH2* in *E. coli*. The concentration of IPTG was 1 mM and the temperature was 30˚C for inducible expression. The left four lanes showed the inducible expression of *SlSAHH2* in supernatant after 0, 2, 4, and 6 h. The right four lanes showed the inducible expression of *SlSAHH2* in inclusion body after 0, 2, 4, and 6 h. The last lane represented for protein marker.

FIGURE S2 The expression of ethylene related genes in flower and IMG fruit. **(A)** Relative expression of *E4, E8, ACO1, ACO3, ACS2* in WT and transgenic flower. **(B)** Relative expression of *E4, E8, ACO1, ACO3, ACS2* in WT and transgenic IMG fruit with diameter less than 1 cm. **(C)** Relative expression of *E4, E8, ACO1, ACO3, ACS2* in WT and transgenic IMG fruit with diameter more than 1 cm. The quantitative PCR data represent mean values for three independent biological replicates (*n* = 3). ^∗^ and ^∗∗^ indicate *P* < 0.05 and *P* < 0.01, respectively.

FIGURE S3 The expression of genes encoding DNA methyltransferases was enhanced in transgenic fruit (OE-5# and OE-6#) at breaker stage. **(A)** Relative expression of *DRM5* in WT and OE lines. **(B)** Relative expression of *DRM7* in WT and OE lines. **(C)** Relative expression of *DRM8* in WT and OE lines. **(D)** Relative expression of *MET1* in WT and OE lines. The quantitative PCR data represent mean values for three independent biological replicates (*n* = 3). ^∗^ and ^∗∗^ indicate *P* < 0.05 and *P* < 0.01, respectively.

TABLE S1 Details of gene primers used in this article.
