# Supplementary figures and images for: Functional Characterization of SlSAHH2 in Tomato Fruit Ripening
Source: Front Plant Sci. 2017 Jul 26;8:1312. doi: 10.3389/fpls.2017.01312 (PMC5526918; doi:10.3389/fpls.2017.01312)

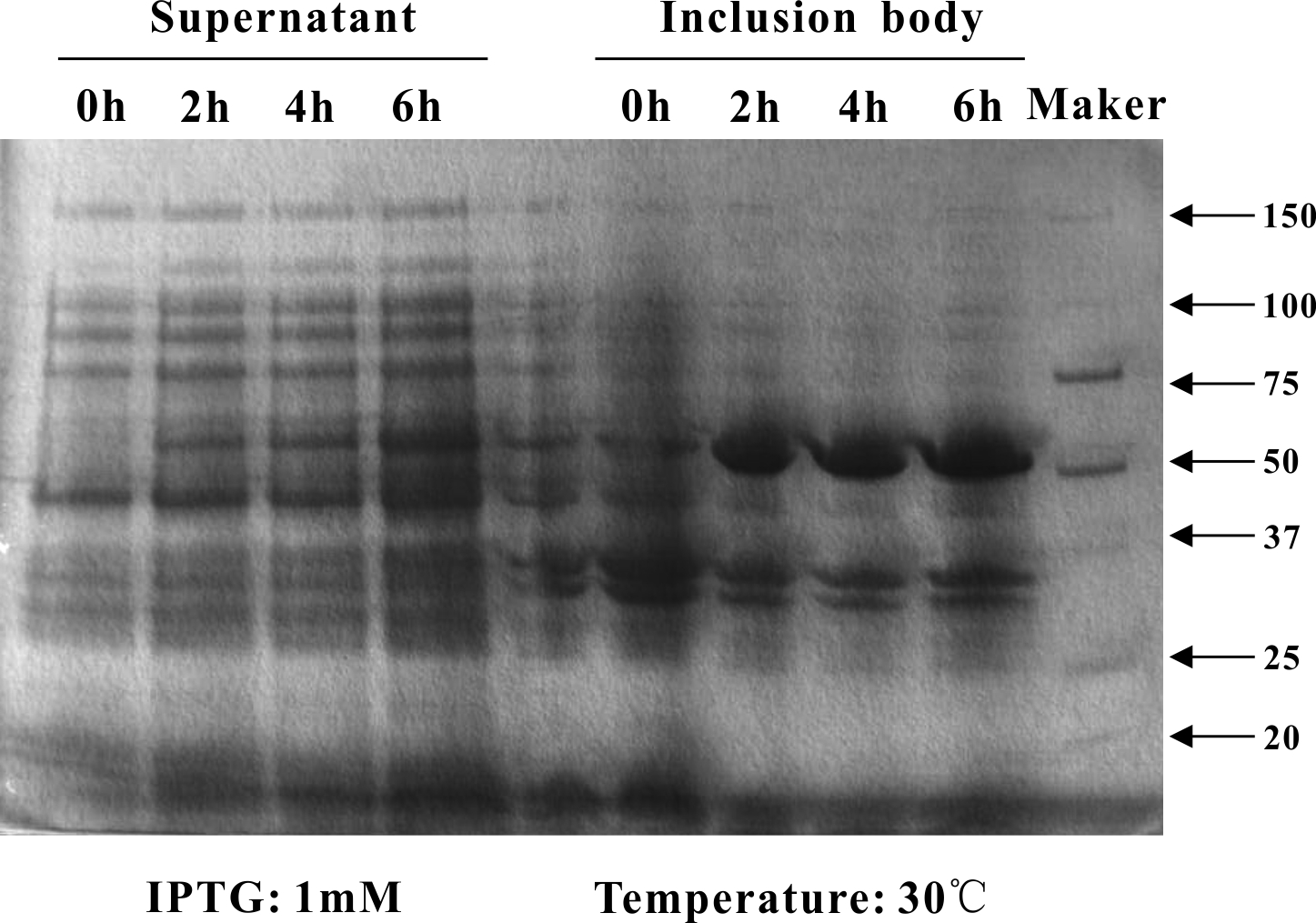

Supplement: FIGURE S1 — Recombinant expression of SlSAHH2 in E. coli. The concentration of IPTG was 1 mM and the temperature was 30˚C for inducible expression. The left four lanes showed the inducible expression of SlSAHH2 in supernatant after 0, 2, 4, and 6 h. The right four lanes showed the inducible expression of SlSAHH2 in inclusion body after 0, 2, 4, and 6 h. The last lane represented for protein marker. [file Image_1.JPEG]

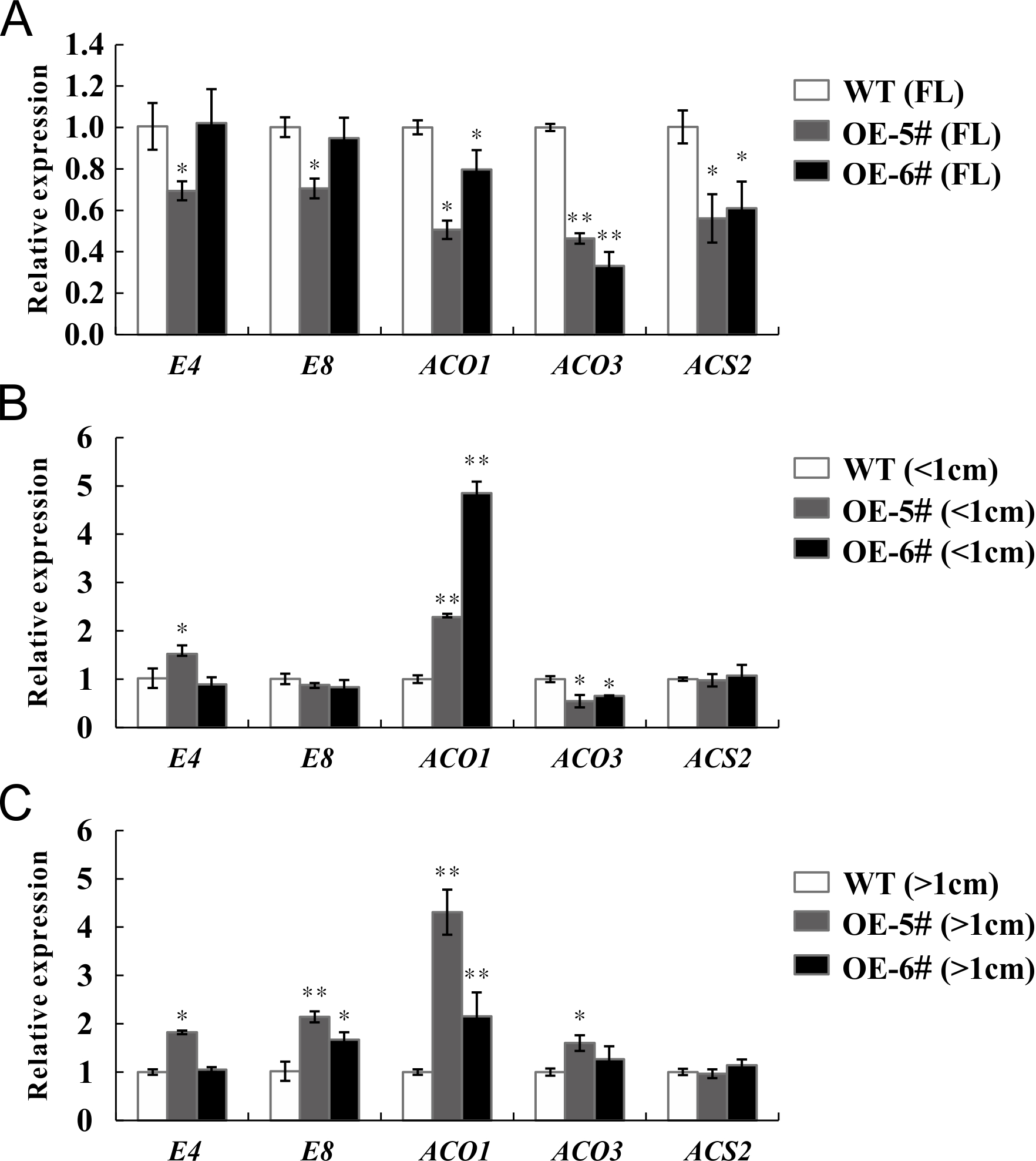

Supplement: FIGURE S2 — The expression of ethylene related genes in flower and IMG fruit. (A) Relative expression of E4, E8, ACO1, ACO3, ACS2 in WT and transgenic flower. (B) Relative expression of E4, E8, ACO1, ACO3, ACS2 in WT and transgenic IMG fruit with diameter less than 1 cm. (C) Relative expression of E4, E8, ACO1, ACO3, ACS2 in WT and transgenic IMG fruit with diameter more than 1 cm. The quantitative PCR data represent mean values for three independent biological replicates (n = 3). ∗ and ∗∗ indicate P < 0.05 and P < 0.01, respectively. [file Image_2.JPEG]

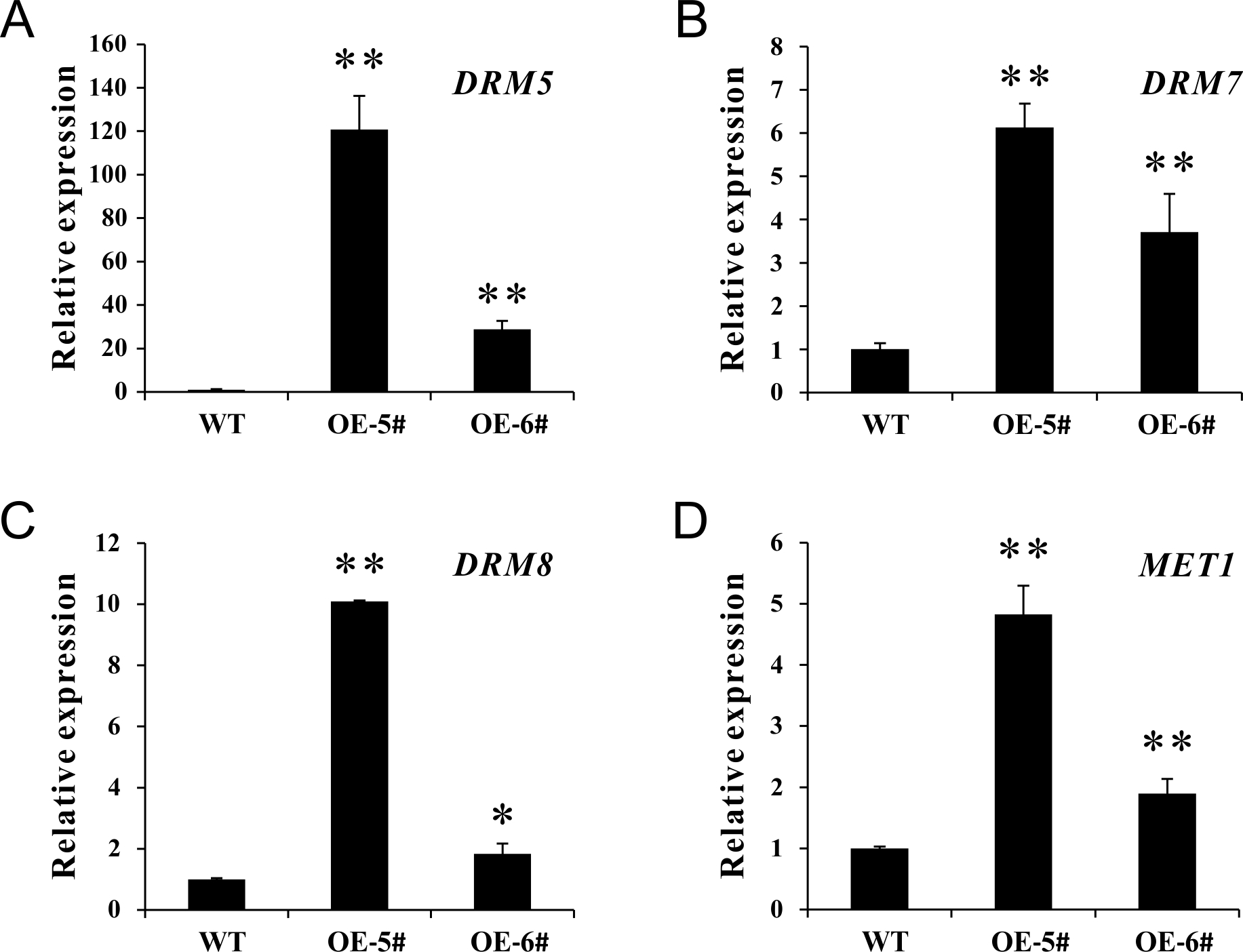

Supplement: FIGURE S3 — The expression of genes encoding DNA methyltransferases was enhanced in transgenic fruit (OE-5# and OE-6#) at breaker stage. (A) Relative expression of DRM5 in WT and OE lines. (B) Relative expression of DRM7 in WT and OE lines. (C) Relative expression of DRM8 in WT and OE lines. (D) Relative expression of MET1 in WT and OE lines. The quantitative PCR data represent mean values for three independent biological replicates (n = 3). ∗ and ∗∗ indicate P < 0.05 and P < 0.01, respectively. [file Image_3.JPEG]
