# Supplementary material for: Functional Characterization of SlSAHH2 in Tomato Fruit Ripening
Source: Front Plant Sci. 2017 Jul 26;8:1312. doi: 10.3389/fpls.2017.01312 (PMC5526918; doi:10.3389/fpls.2017.01312)
Supplement: TABLE S1 — Details of gene primers used in this article. [file Table_1.DOC]

**Table S1. Details of gene primers used in this article**

| Name | Sequence (5’-3’) | Description |  |
| --- | --- | --- | --- |
| SlSAHH1  SlSAHH2  SlSAHH3  SAHH2 | F: AAGGAGACAGACAAAGTGAGACTG  R: CAAAGAAAATCATAAACCAAAACCC  F: GACGCTCACAGTGGAACAACGA  R: TCAACTTTCCAGCAAAATATCCCC  F: TACTGAGGGAAGACAATTTGAC  R: CAAAACATGCACAAATATATCCC  F: CTCTCAGATCTCATCTTAAACCC  R: CAAACACATTTAGGTCAACTTTCC  F: GGATCCGCTCTACTCGTTGAGAAGACCAC  R: GAGCTCGTTCCACTGTGAGCGTCTCTTCC  F: TGTCCCTATCTACGAGGGTTATGC  R: AGTTAAATCACGACCAGCAAGAT  F: AGGGTAACAACAGCAGTAGCA  R: CCCAACCTCCGTCTTCAC  F: GGCACCATTCAACATACCG  R: CTTTCACCGAAGAAGCACG  F: ACAAACAGACGGGACACGAA  R: CTCTTTGGCTTGAAACTTGA  F: CAAGCAAGTTTATCCGAAAT  R: CATTAGCTTCCATAGCCTTC  F: GAAAGAGTTGTTATGGCTGGTG  R: GCTGGGTAGTATGGTGAAGGT  F: ATGCAGCACCATCAACACAT  R: CTCCAAATTCAAAGCATCCA  F: AACGGACCACAATCTTGAC  R: CTGCTCGGAGTCTGAACC  F: ACTTTCTGTTCTTTGTGATGCT  R: TTGGATGCTTCTTGCTGGTAG  F: GCCAAATCAAGCAATGATGA  R: TCGCAACCATACAGACCATT  F: AGAGAACGATGCATGGAGGTTTGT  R: ACTGGCTCAGGAAATTGGCAATGG  F: TGTGGTCCAGAAGCATCGG  R: TCTTCGGACAATTGCAGAAACT  F: AACAGGGTGACTAGAGACGGACT  R: GATGGCACTAAGAGAGTTACTTTGTG  F: GTTTTATGCTTTTGCTGTGGC  R: TGTCCCCCATAGGCATTGTA  F: GGGTTGTCCGAGCGATGA  R: GCTTTTGGGCCAATACGTAGA | Expression pattern analysis  Expression pattern analysis  Expression pattern analysis  Construction of pLP100-35S-SAHH2  Construction of pET28(a)-SAHH2  Housekeeping gene  Real-time PCR  Real-time PCR  Real-time PCR  Real-time PCR  Real-time PCR  Real-time PCR  Real-time PCR  Real-time PCR  Real-time PCR  Real-time PCR  Real-time PCR  Real-time PCR  Real-time PCR  Real-time PCR | |
|  |
| rSAHH2 |
|  |
| SlActin |
|  |
| E4 |
|  |
| E8 |
|  |
| ACO1 |
|  |
| ACO3 |
|  |
| ACS2 |
| RIN  AP2a  TAGL1  CNR  NOR  DRM5  DRM7  DRM8  MET1 |
